# Mitochondrial Reactive Oxygen Species Modulate Mosquito Susceptibility to *Plasmodium* Infection

**DOI:** 10.1371/journal.pone.0041083

**Published:** 2012-07-18

**Authors:** Renata L. S. Gonçalves, Jose Henrique M. Oliveira, Giselle A. Oliveira, John F. Andersen, Marcus F. Oliveira, Pedro L. Oliveira, Carolina Barillas-Mury

**Affiliations:** 1 Laboratory of Malaria and Vector Research, National Institute of Allergy and Infectious Diseases, National Institutes of Health, Rockville, Maryland, United States of America; 2 Laboratório de Bioquímica de Resposta ao Estresse, Instituto de Bioquímica Médica, Universidade Federal do Rio de Janeiro, Rio de Janeiro, Brazil; 3 Laboratório de Bioquímica de Artrópodes Hematófagos, Instituto de Bioquímica Médica, Universidade Federal do Rio de Janeiro, Rio de Janeiro, Brazil; 4 Laboratório de Inflamação e Metabolismo, Instituto Nacional de Ciência e Tecnologia de Biologia Estrutural e Bioimagem, Universidade Federal do Rio de Janeiro, Rio de Janeiro, Brazil; 5 Instituto Nacional de Ciência e Tecnologia em Entomologia Molecular, Rio de Janeiro, Brazil; Johns Hopkins Bloomberg School of Public Health, United States of America

## Abstract

**Background:**

Mitochondria perform multiple roles in cell biology, acting as the site of aerobic energy-transducing pathways and as an important source of reactive oxygen species (ROS) that modulate redox metabolism.

**Methodology/Principal Findings:**

We demonstrate that a novel member of the mitochondrial transporter protein family, *Anopheles gambiae* mitochondrial carrier 1 (AgMC1), is required to maintain mitochondrial membrane potential in mosquito midgut cells and modulates epithelial responses to *Plasmodium* infection. AgMC1 silencing reduces mitochondrial membrane potential, resulting in increased proton-leak and uncoupling of oxidative phosphorylation. These metabolic changes reduce midgut ROS generation and increase *A. gambiae* susceptibility to *Plasmodium* infection.

**Conclusion:**

We provide direct experimental evidence indicating that ROS derived from mitochondria can modulate mosquito epithelial responses to *Plasmodium* infection.

## Introduction

Malaria is a deadly disease caused by *Plasmodium* parasites and results in more than half a million deaths every year, mainly of African children [1]. *Plasmodium* is transmitted by anopheline mosquitoes, and the establishment of this protozoan parasite in the insect vector can be greatly hindered by mosquito antiplasmodial responses [2], [3]. To complete their development in the mosquito, *Plasmodium* parasites have to traverse the midgut epithelium and avoid destruction by the mosquito immune system. *Plasmodium* invasion causes irreversible damage that leads to apoptosis of invaded midgut cells [4]. Midgut epithelial cells respond to *Plasmodium* ookinete invasion by inducing expression of nitric oxide synthase (NOS) and heme peroxidase 2 (HPX2), two enzymes that mediate nitration [5], [6]. As ookinetes emerge from the midgut, they come in contact with components of the mosquito complement system present in the hemolymph, such as thioester-containing protein 1 (TEP1), and suffer major parasite losses during the ookinete to oocyst transition [7]. Recent studies revealed that HPX2 potentiates NOS toxicity by promoting midgut nitration, and this reaction also modifies ookinetes as they traverse the epithelium, making them “visible” to the mosquito complement system [6]. The nitration response also requires the participation of NADPH oxidase 5 (Nox5) as a source of hydrogen peroxide for HPX2 to be active [6].

Previous studies have shown that reactive oxygen species (ROS) levels are elevated in an *Anopheles gambiae* strain refractory to *Plasmodium* infection [8] and that ROS are required for mosquitoes to mount effective immune responses against bacteria and *Plasmodium* [9]. The classical source of ROS associated with the immune response is NADPH oxidase/Nox or Duox family of proteins present in the epithelia of both vertebrates [10], [11], [12] and invertebrates [6], [13], [14]. However, recent studies in vertebrates revealed that other cellular sources of ROS, such as mitochondria, play an important role in macrophage-mediated immunity [15], [16]. There is increasing evidence that mitochondria participate in innate immune responses [17] against viral and bacterial infections [15], [18] and that mitochondrial ROS are important activators of the host immune response to infection [19], [20].

Mitochondria are the main cellular energy-transducing site and also a significant source of ROS [21]. In the energy conversion process, energy rich substrates are oxidized, donating electrons to a group of proteins located in the mitochondrial inner membrane, known as mitochondrial complexes (I to IV). The electrons are sequentially passed through the mitochondrial complexes, and concomitant to this electron flow, some complexes pump protons (H^+^) into the mitochondrial intermembrane space; giving rise to a membrane potential between the mitochondrial membranes. Mitochondrial ATP production is achieved through oxidative phosphorylation, a process where the F_1_F_0_-ATP synthase complex, uses the energy of the electrochemical gradient to phosphorylate ADP to ATP. Under normal circumstances, a small portion of electrons leak from the electron transport system, giving rise to ROS [21]. ROS production is highly controlled by the mitochondrial membrane potential (ΔΨ_m_), so that small changes in this parameter can drastically affect ROS generation [22]. When the mitochondrial membrane potential is dissipated before it can be used to generate ATP, the energy from the electron flow is uncoupled from ATP production. Several lines of evidence indicate that mild uncoupling, either pharmacological [23] or physiological [24], [25], [26], reduces ROS generation.

To assess mitochondrial function, cellular oxygen consumption rates can be measured and modulated by experimental addition of substrates and/or inhibitors [27]. State 3 respiration is defined as the ADP-stimulated oxygen consumption and reflects the phosphorylation-dependent respiration; while the phosphorylation-independent oxygen consumption (state 4), can be evaluated by adding oligomycin, an inhibitor of ATP synthase. The respiratory control ratio (RCR) is defined as the ratio between state 3/state 4 respiration and reflects the integrity of the inner mitochondrial membrane. It is also possible to measure the maximal respiratory capacity by the addition of the proton ionophore FCCP, which dissipates the mitochondrial membrane potential and increases respiration. FCCP-treated mitochondria exhibit low membrane potential resulting in oxidative phosphorylation uncoupling and impaired mitochondrial ATP synthesis.

The uncoupling proteins (UCP) and the adenine nucleotide translocators (ANT) are members of the solute carrier family 25 (SLC25) [28], a family of mitochondrial proteins [29] that is responsible for the transport of metabolites and protons. UCP and ANT are known to promote mitochondrial uncoupling and reduce ROS production [24], [30], [31], [32]. Furthermore, disruption of UCP2 in a murine model confers resistance to *Toxoplasma gondii* infection through a mechanism that involves increased ROS generation [15]. Members of SLC25 are structurally and functionally related [28] and conserved across species, but they are poorly explored in insects. In this work we identified a new member of the SLC25 family in *A. gambiae*, the mitochondrial carrier 1 (AgMC1), which affects mitochondrial coupling, ROS generation and susceptibility to *Plasmodium* infection.

## Materials and Methods

### Ethics Statement

Public Health Service Animal Welfare Assurance #A4149 01 guidelines were followed according to the National Institutes of Health (NIH) Office of Animal Care and Use (OACU). This study was approved by the NIH Animal Care and User Committee (ACUC), under the NIH animal study protocol (ASP) ID ASP LMVR5.

### Insects

*A. gambiae* (G3 and L3-5 strain) and *Aedes aegypti* (Red eyes strain) [33] mosquitoes were reared at 28°C, 75% humidity, under a 12-h light/dark cycle and maintained on a 10% sucrose solution during adult stages.

### Midgut Respiration

Respiration assays were done following the procedures first described by Chance and Williams, 1955 [27] and recently adapted to work with mosquitoes [33], [34]. Midguts from 30 adult *A. gambiae* females were dissected in isolation buffer consisting of 250 mM sucrose, 5 mM Tris-HCl, 2 mM EGTA, 1% (w/v) fatty acid-free bovine serum albumin (BSA), pH 7.4 and placed on an oxygraph chamber (Hansatech Instruments Ltd, England) with respiration buffer (120 mM KCl, 5 mM KH_2_PO_4_, 3 mM Hepes, 1 mM EGTA, 1 mM MgCl_2_, and 0.1% fatty acid-free BSA, pH 7.2) supplemented with 0.0025% digitonin. After 1 min of equilibration, both NAD^+^-linked substrate (10 mM pyruvate +10 mM proline) and FAD^+^-linked substrate (10 mM succinate) were added to the chamber. Subsequently, 1 mM ADP was added to induce the state 3 respiratory state. State 4-like (oligomycin-stimulated) and uncoupled maximal respiratory rates (carbonyl cyanide p-trifluoromethoxy-phenylhydrazone; FCCP-stimulated) were measured upon addition of 7 µg of oligomycin and up to 7 µM FCCP, respectively. The RCR was calculated by dividing the state 3 respiratory rates to those obtained by the oligomycin-induced state 4 respiratory rates (2 min after oligomycin addition in the case of *Anopheles gambiae* and 5–6 minutes in the case of *Aedes aegypti*). Oxygen (O_2_) consumption was recorded in an oxygraph fitted with a Clark-type electrode in a water-jacketed chamber (Oxytherm, Hansatech Instruments, Norfolk, England) for *Anopheles gambiae* and in a high-resolution oxygraph (O2k – Oroboros Inc., Austria) for *Aedes aegypti.* For all experiments, temperature was maintained at 27.5°C. The respiratory inhibitors antimycin A, and cyanide were all capable of completely inhibiting the midgut O_2_ consumption. All mosquitoes used for the respiration assay were 4–7 days old. All mitochondrial respiration results represent the averaged oxygen consumption of at least six different midgut preparations analyzed in three independent experiments.

### Infection of Mosquitoes with *Plasmodium*

Mosquitoes were infected with *P. berghei* (GFP-CON transgenic 259cl2 strain) [35] by feeding them on anesthetized infected BALB/c mice. Mouse infectivity was established by determining the parasitemia and by performing an *in vitro* exflagellation assay, as described previously [36]. In all the studies, mouse parasitemia was 4–5% and the number of exflagellations per field was 1–2 under a 40× objective. Blood-fed mosquitoes were kept at 21°C and 80% humidity. Infection phenotypes were determined 7–8 days post infection by fixing for 30 min in 4% formaldehyde and mounting the midguts in glass slides with VectaShield (Vector Laboratories, Burlingame, CA). *Aedes aegypti* females (Red Eye strain) were fed with *P. gallinaceum*-infected chickens (∼5% parasitemia). 7 days after infection midguts were dissected and oocysts were counted under a light microscope after 2% mercurochrome staining. The distribution of the number of oocysts in individual mosquitoes were compared using the non-parametric Kolmogorov-Smirnov (KS) test, the median levels of infection were compared using the Mann-Whitney test and the prevalence of infection using the Chi-square test.

### Antibiotic Treatment

A solution of penicillin (100 units/ml) and streptomycin (0.1 mg/ml) (Sigma Aldrich, St. Louis, MO) in 10% sugar solution was orally administered to mosquitoes on the first day post emergence. At the following day, they were injected with dsRNA from the gene of interest and kept with antibiotic solution in water (a sugar cube was provided separately) until 2 days post injection when the experiments were performed.

### Cloning and Sequencing of AgMC1 cDNA

RNA was extracted from midguts of sugar-fed mosquitoes with Trizol (Invitrogen, San Diego, CA) according to the manufacture’s protocol. First-strand cDNA was synthesized using QuantiTect Reverse Transcriptase (QIAGEN Inc., Valencia, CA). The full-length AgMC1 sequence (1498 bp) was amplified using the following primers: F-TGCACTCGT TCTATTTTCTACTGC and R-CGAAGTGGAGGAACTGCTACTAA and cloned with TOPO TA cloning kit (Invitrogen, San Diego, CA) following standard procedures. Primers were designed based on the cDNA sequence predicted by the bioinformatics annotation of the AgMC1 gene in the *A. gambiae* genome sequence (GenBank accession No. AGAP001297-PA).

### dsRNA Synthesis

A 218-bp fragment of the lacZ gene was amplified using the primers (5′ to 3′) F-GAGTCAGTGAGCGAGGAAGC and R-TATCCGCTCACAATTCCACA and cloned into the pCRII-TOPO vector. T7 promoters were incorporated onto this fragment by amplifying the cloned insert using the following primers: M13F-GTAAAACGACGGCCAGT and M13R-CTCGAGTAATACGACTCACTATA GGGCAGGAAACAGCTATGAC. The PCR product was used as a template to synthesize dsRNA in vitro using a MEGAscript RNAi kit (Ambion, Austin, TX). dsRNA was loaded to an RNA purification column supplied by the kit, eluted with water and concentrated to 3 µg/µl using a Microcon YM-100 filter (Millipore Corporation, Billerica, MA). A similar cloning strategy was used for the AgMC1. The cDNA from AgMC1 was used to generate dsRNA templates of 698 bp using the following primers: AgMC1 (5′ to 3′) F-TAATACGACTCACTATAGGGAGCAAGCGTCCCCTACACT and R-TAATACGACTCACTATAGGGCGTTTTGACCACGTCGAAC. For A. aegypti MC1 dsRNA synthesis a nested PCR was performed using the external primers (5′ to 3′) F- GCCTTGCCCACTACAGTGAT and R-gggtccgaactgaactcatc and the internal primers fused with the T7 promoters F- TAATACGACTCACTATAGGGAACGATTGTGAATCCGTTGG and R- TAATACGACTCACTATAGGGTATAAGTGCGCCCTGATCCT (GenBank accession No. AAEL001329-RA).

### Gene Silencing

*A. gambiae* and *A. aegypti* female mosquitoes were injected with 69 nl and 138 nl, respectively, of a 3-µg/µl solution of dsRNA from the gene of interest at 1–2 days post emergence. Control mosquitoes were injected with dsLacZ. Two days later, females from both groups (dsLacZ, dsAgMC1 or AaMC1) were used. The distributions of the number of oocysts in individual mosquitoes were compared using the non-parametric Kolmogorov-Smirnov (KS) test, the median levels of infection were compared using the Mann-Whitney test and the prevalence of infection with the Chi-square test. The effect of AgMC1 and AsMC1 silencing on *P. berghei* and *P. gallinaceum*, respectively, were each confirmed in two independent experiments that were merged.

### Quantitation of Gene Expression

RNA extraction and cDNA synthesis were performed as described above. Gene expression was assessed by SYBR green quantitative real-time PCR (qPCR) (DyNAmo HS; New England Biolabs Inc., Ipswich, MA) in a Chromo4 system (Bio-Rad Laboratories, Hercules, CA). PCR involved an initial denaturation at 95°C for 15 min, followed by 44 cycles of 10 s at 94°C, 20 s at 56°C, and 30 s at 72°C. Fluorescence readings were taken at 72°C after each cycle. A final extension at 72°C for 5 min was completed before deriving a melting curve (70°C–95°C) to confirm the identity of the PCR product. qRT-PCR measurements were made in triplicate. Relative quantitation results were normalized with *A. gambiae* ribosomal protein S7 as an internal standard and analyzed by the 2-ΔΔCt method [37]. The primers used to evaluate gene expression were AgMC1, F-TTG TGAAATCAAACCCAGCA and R-CCACGTCCAGTGGAGT CATA. S7, F-GGCGATCAT CATCTACGTGC and R-GTAGCTGCTGCAAACTTCGG. AaMC1, F- TTCATGACCCCGCTAGATGT and R- CCCTCGTGGTGACTGATTTT. RP-49, F-GCTATGACAAGCTTGCCCCCA and R-TCATCAGCACCTCCAGCT. AgDuox, F-CAAGGACAAGGACGGAAGAA and R-TCGCACATGTCGAAAATGAT. AgNox5, F-TGTCTTCACGCAGAAGGATG and R-ACCCACGGGATAGTTCACTG.

### Midgut Hydrogen Peroxide (H_2_O_2_) Production

H_2_O_2_ production was assessed by monitoring resorufin fluorescence due to the oxidation of 5 mM Amplex Red (Invitrogen) in the presence of 1.0 unit/ml of commercial horseradish peroxidase (HRP) (Sigma). Five midguts, from antibiotic treated mosquitoes, were dissected in 10 mg/ml 3-amino-1,2,4-triazole in PBS (AT) (MP Biomedicals, LLC, Solon, OH) and incubated at room temperature and dim light in AT solution with Amplex Red and HRP for 30 min. Amino-triazole was used to inhibit H_2_O_2_ consumption by midgut catalase. Fluorescence intensity was measured in the supernatant in a spectrofluorometer plate reader (SpectraMax gemini XPS; Molecular Devices) operating at excitation and emission wavelengths of 530 nm and 590 nm, respectively. Background fluorescence generated as unspecific Amplex Red oxidation by the midgut in the absence of HRP was subtracted. After each experiment, a standard curve of reagent grade H_2_O_2_ (Merck, Darmstadt, Germany) was performed.

### ROS Production in Midgut Cultures

Midguts of sugar-fed mosquitoes were dissected in PBS and incubated at room temperature in dim light in RPMI supplemented with 10% FBS containing 5 µM of the intracellular ROS-sensitive dye DHE (Molecular Probes, Invitrogen) for 20 min [38]. Midguts were washed with fresh media, mounted in glass slides, and immediately photographed under a fluorescence microscope (Leica, Solms, Germany). The midgut staining presented is a representative image of 13 midguts analyzed for each treatment (LacZ or AgMC1). These results were confirmed in two independent experiments.

### Midgut Mitochondrial Staining

Females were fed with a solution containing PBS + MitoTracker Red (CM-H2-XRos; Molecular Probes) (20 mM) + ATP (0.5 mM), pH 7. One hour after feeding, the midguts were dissected in PBS, fixed with paraformaldehyde (4%) for 40–60 minutes in the dark, washed twice with PBS, permeabilized with Triton X-100 (0.01%) for 3–5 min, and washed again. F-actin was stained with Phalloidin-Alexa 488 (Molecular Probes) (165 nM) diluted in PBS + BSA 1% at room temperature for 20 min followed by two washings with PBS. Finally, the midguts were transferred to glass slides and mounted with VectaShield with DAPI (Vector Laboratories). Images were acquired on a SP2 confocal microscope (Leica). The staining pattern was confirmed in three independent experiments.

### Early Oocyst Count

Assessment of early oocyst numbers in the midgut was done 48 h post-infection, using immunofluorescence staining with anti-Pbs21 antibodies to detect the parasites. Midguts were dissected in ice-cold PBS, fixed for 30 s in 4% paraformaldehyde and returned to ice-cold PBS to stop the fixation. The midgut was opened longitudinally to remove the blood and the epithelia was carefully cleaned, fixed overnight with 4% paraformaldehyde at 4°C and washed twice with PBS. Midguts were permeabilized and blocked for 2 h at room temperature with PBT (1% BSA, 0.1% Triton X-100 in PBS) under gentle shaking. Midguts were incubated with primary Ab (anti-Pbs21; mouse; 1∶300 dilution) overnight at 4°C followed by three washing steps of 20 min with PBT at room temperature. The incubation with the secondary Ab (anti-mouse IgG-Cy3; 1∶1000) carried out for 2 h at room temperature. The samples were then washed as described above and mounted using VectaShield. The number of early oocysts was scored in a Leica fluorescence microscope under the 40x objective.

### Molecular Modeling of AgMC1

The sequence of AgMC1 was submitted for automated molecular modelling using the Phyre protein fold recognition server (http://www.sbg.bio.ic.ac. uk/∼phyre/), which identifies homologs with known structures using PSI-BLAST and generates a homology-based model using the three-dimensional coordinates and the amino acid sequence alignment information [39].

## Results

### Characterization of the Mitochondrial Carrier AgMC1

**Figure 1:**
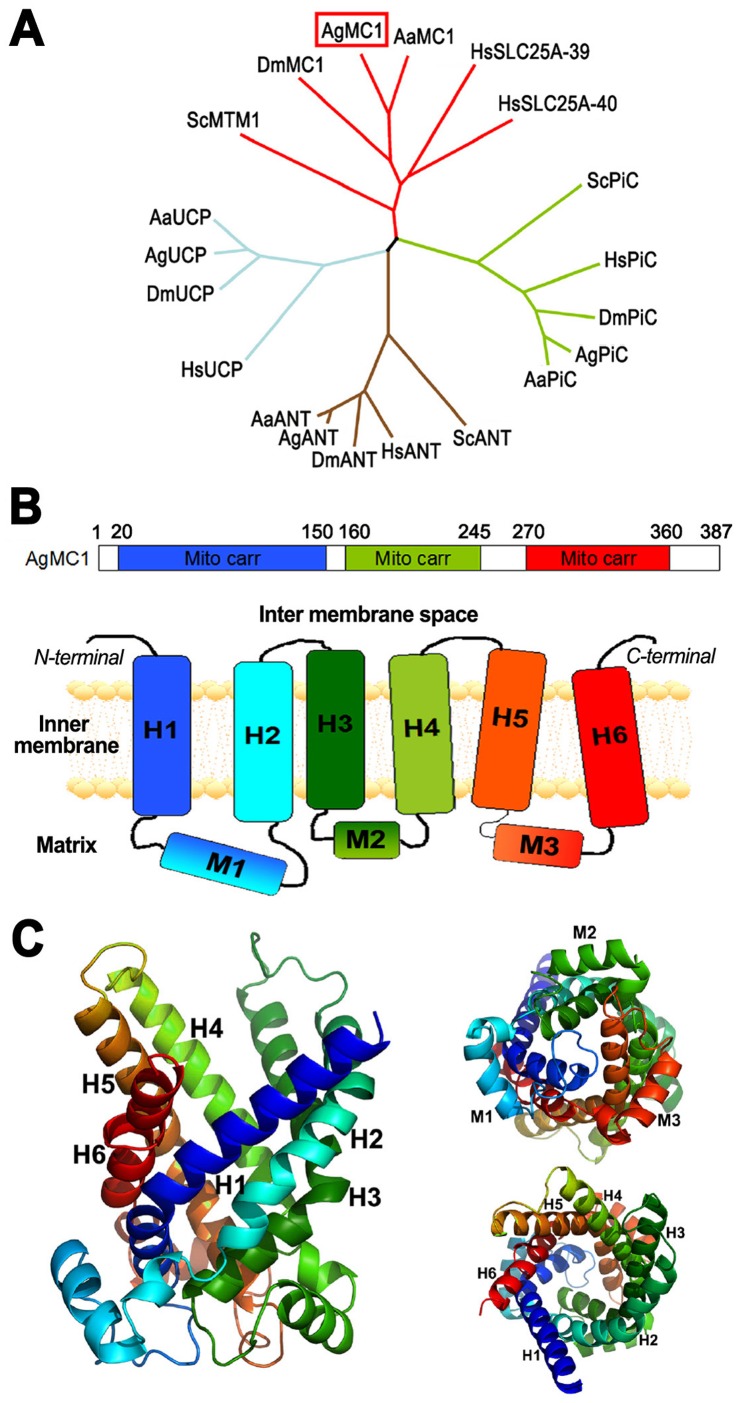
*Anopheles gambiae* mitochondrial carrier 1 (AgMC1) phylogeny and predicted structure. (A) Phylogenetic tree based on the sequence alignment of the deduced amino acid sequence of AgMC1 (AGAP001297-PA) and the putative mitochondrial carriers from *A. aegypti* (AaMC1), *D. melanogaster* (DmMC1), human mitochondrial carriers HsSLC25A-39 and SLC25A-40 and yeast manganese trafficking factor for mitochondrial (ScMTM1); uncoupling proteins from humans (HsUCP), *A. gambiae* (AgUCP), *A. aegypti* (AaUCP) and *D. melanogaster* (DmUCP); putative adenine nucleotide translocators (ANT) from humans (HsANT, SLC25A6), yeast (ScANT), *A. gambiae* (AgANT) *A. aegypti* (AaANT), and *D. melanogaster* (DmANT); putative phosphate carriers (PiC) from humans (HsPiC, SLC25A3), yeast (ScPiC), *A. gambiae* (AgPiC), *A. aegypti* (AaPiC,) and *D. melanogaster* (DmPiC). Sequence alignments and accession numbers are included in Fig. S1. (B) Schematic representation of the AgMC1 protein sequence coding for three mitochondrial carrier domains (mito carr, top panel) highlighted in blue, green and red, and of the predicted secondary structure (bottom panel), consisting of six transmembrane domains (H1 to H6), three matrix domains, (M1 to M3), and cytosolic domains. (E) Predicted tertiary structure based on the amino acid sequence of the AgMC1 based on the known structure of bovine ADP/ATP adenine nucleotide translocator. Ribbon diagram of the predicted structure of AgMC1 from a lateral view (left), or viewed from either the matrix (top right) or intermembrane space side of the mitochondrial membrane (bottom right).

Previous studies showed that expression of some genes related to the mitochondrial electron transport chain are induced in the *Plasmodium*-resistant *A. gambiae* (L3-5) refractory mosquito strain [8], [34], and that this strain has a higher rate of mitochondrial electron leak, suggesting that differences in mitochondrial metabolism affect mosquito susceptibility to *Plasmodium* infection [34]. We decided to investigate the potential role of AgMC1 in the mosquito redox balance and susceptibility to infection because some members of the solute carrier family are known to promote mitochondrial uncoupling and reduce ROS production [24], [30]–[32] and the *AgMC1* gene is located in Chr 2 division 7B, a chromosomal region in *A. gambiae* that has been associated with the refractory phenotype [40]. A phylogenetic tree was built based on the sequence alignment of the deduced amino acid sequence of AgMC1 (accession number AGAP001297-RA) with mitochondrial transporters from different species (Figure 1A, Figure S1 and Table S1). AgMC1 has the highest homology to putative ortholog genes in *Aedes aegypti* (87% homology) and *Drosophila melanogaster* (72%), and clusters with the mammalian SLC25 family members 39 (60%) and 40 (64%) and the yeast manganese trafficking protein 1 (MTM) (48%) [41](37). SLC25 transporters share three conserved domains of approximately 100 amino acids that are the signature feature of mitochondrial carriers (Figure 1B, top). The predicted secondary structure of AgMC1 is shown as a schematic diagram in Figure 1B (bottom) and follows the same color scheme (blue, green, and red) as the linear diagram. It consists of six predicted transmembrane domains (H1-H6) joined together by the mitochondrial matrix (M1–M3) and cytosolic domains. Each mitochondrial carrier domain comprises a set of two transmembrane helixes and one matrix loop.

A molecular model of the AgMC1 three-dimensional structure was constructed using the automated Phyre fold recognition server (Figure 1C). The strongest structural match of AgMC1 was with the bovine ADP/ATP carrier protein or adenine nucleotide translocators (ANT) [42]. All the structural elements of the ADP/ATP carrier protein were present in AgMC1, including six transmembrane α-helices (H1–H6) and three matrix loop elements (M1–M3) known to be located on the matrix side of the inner membrane. The AgMC1 sequence differs from that of the ADP/ATP carrier in having a 29-amino acid residue insertion in matrix loop 1 (M1) and a 14-residue insertion in matrix loop 3 (M3), while matrix loop 2 (M2) is similar in length to the ADP/ATP carrier. It is not possible to predict the natural AgMC1 ligand based on the molecular model, but the sequence clearly lacks the RRRMMM hexapeptide signature sequence present on the C-terminal end of transmembrane helix 5 (H5) of ADP/ATP transporters. However, the binding depression of AgMC1, like that of the ADP/ATP carrier, is also lined with a variety of charged and polar amino acid side chains.

### AgMC1 Silencing Increases *Plasmodium* Infection

**Figure 2:**
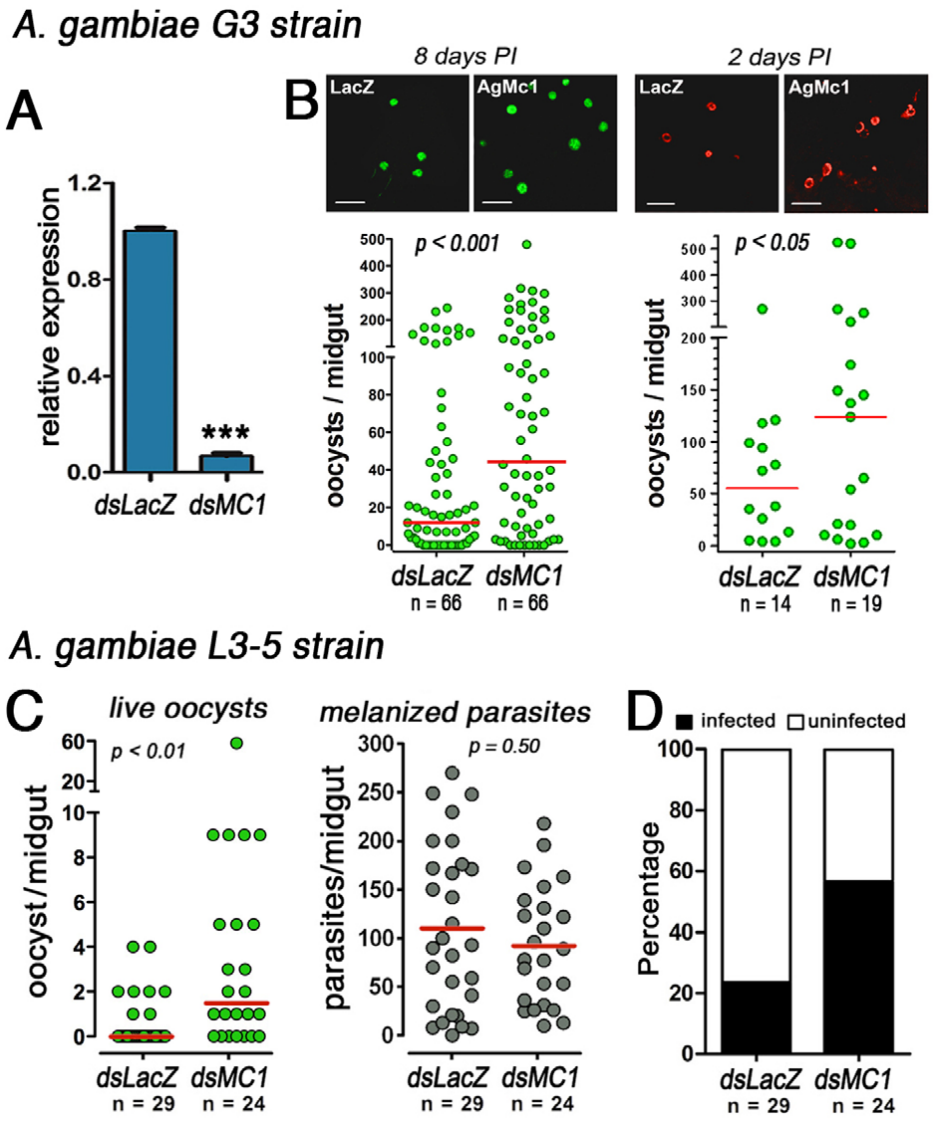
Effect of AgMC1 silencing on *A. gambiae* susceptibility to *P. berghei* infection. (A) AgMC1 midgut mRNA levels in sugar-fed *A. gambaie* G3 females 2 days after injection of dsLacZ (control) or dsAgMC1. Effect of AgMC1 silencing on the number of *Plasmodium berghei* oocysts in midguts dissected (A) 8 days or (B) 2 days post feeding. Effect of AgMC1-silencing on the number of live (green dots, left panel) or melanized (black dots, right panel) (C) and prevalence (D) of *P. berghei* infection in *A. gambiae* refractory (L35 strain) females 7 days post-infection. Medians are indicated by the red lines and distributions were compared using the Kolmogorov-Smirnov test. (***indicates *P*<0.001; Student’s t test).

The effect of AgMC1-silencing on *Anopheles gambiae* G3 susceptibility to *Plasmodium berghei* infection was investigated. AgMC1 expression was efficiently silenced and 2 days post-injection midgut mRNA levels decreased by 93% relative to the dsLacZ control group (Figure 2A). AgMC1 silencing significantly increased the number of developing oocysts present 8 days post-infection (PI) (*P*<0.001) (Figure 2B, left panel). The effect of AgMC1 silencing on infection was also evaluated at an earlier time (2 days PI) to determine whether this treatment enhanced survival early or late stages of the parasite in the mosquito. The number of early oocysts was already significantly higher in AgMC1 silenced females (Figure 2B, right panel), indicating that early stages of the parasites survive better when AgMC1 expression is reduced (P<0.05).

The *A. gambiae* (L3-5 strain) is refractory (R) to infection with *P. berghei* and parasites are melanized as soon as they complete invasion and come in contact with mosquito hemolymph. AgMC1 silencing also increased the number of oocysts that developed in the R strain (*P*<0.01) (Figure 2C, left panel), as well as the prevalence of infection, from 23% to 56% (*P*<0.001) (Figure 2D). However, the effect of AgMC1 silencing on the refractory phenotype was partial, as most parasites were still melanized (Figure 2C, right panel). Because in the R strain parasites are killed and melanized during the ookinete to oocyst transition, these findings also indicate that AgMC1 silencing promotes survival of early stages of *Plasmodium* in the mosquito midgut.

### Participation of AgMC1 in Generation in the Midgut

**Figure 3:**
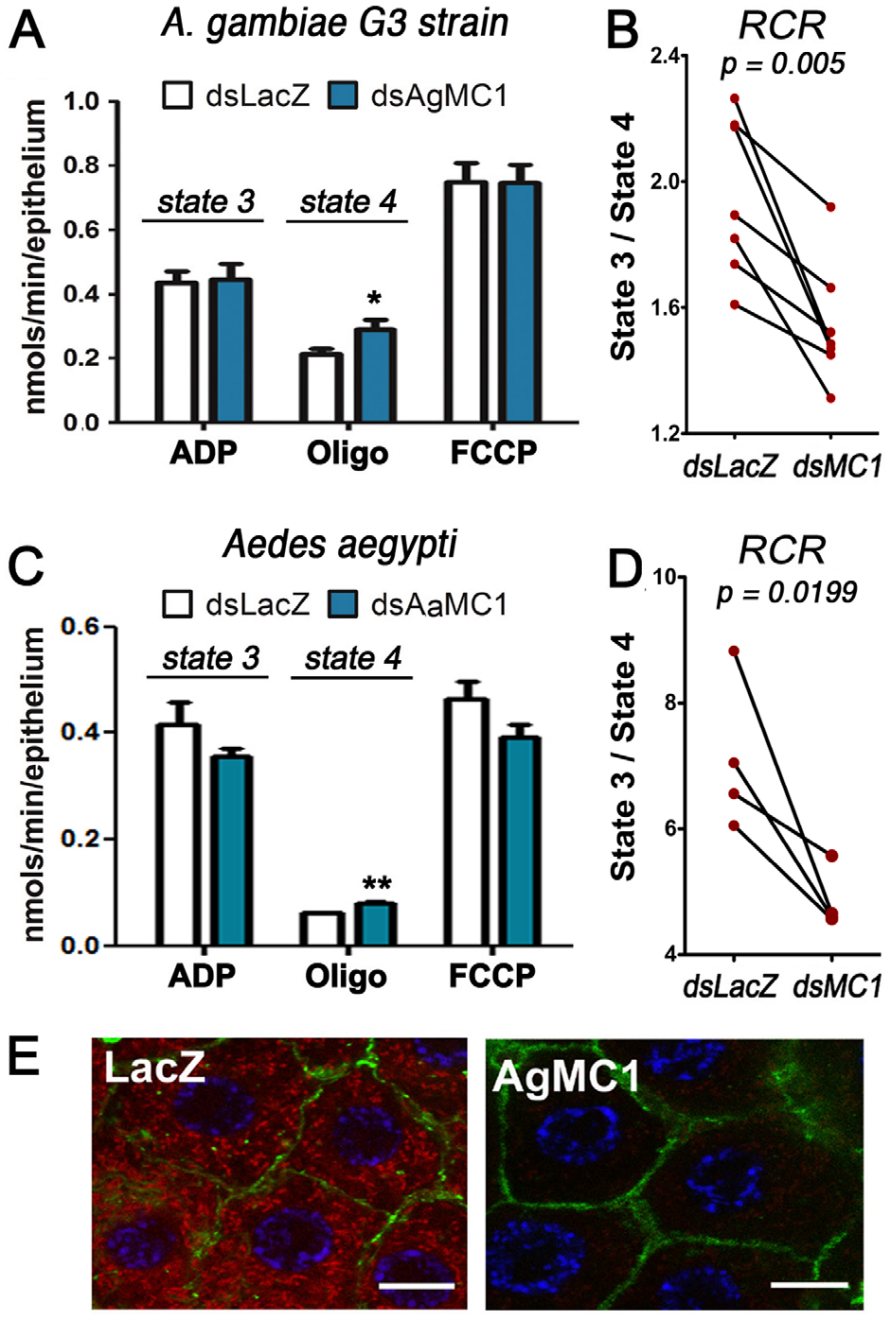
Effect of AgMC1 silencing on midgut mitochondrial respiration and coupling. (A) Oxygen consumption of midguts from sugar-fed *A. gambiae* females injected with dsLacZ or dsAgMC1 after the addition of ADP, oligomycin (oligo), or carbonyl cyanide p-trifluoromethoxyphenylhydrazone (FCCP). (B) RCR from paired groups of *A. gambiae* midguts. The *A. gambiae* respiration data represent seven biological replicates from three independent experiments. (C) Oxygen consumption of midguts from sugar-fed *A. aegypti* females injected with dsLacZ or dsAaMC1 after the addition of ADP, oligomycin (oligo), or carbonyl cyanide p-trifluoromethoxyphenylhydrazone (FCCP). (D) RCR from paired groups of *A. aegypti* midguts. The *A. aegypti* respiration data represent four biological replicates from two independent experiments. (E) Effect of AgMC1 silencing midgut on mitochondrial membrane potential. Confocal analysis of control (dsLacZ) and silenced (dsAgMC1) midguts stained with MitoTracker Red (CM-H_2_-XRos) to evaluate membrane potential. Nuclei were stained with DAPI (blue) and F-actin with green phalloidin. Scale bar  = 8 µm. (*and **indicate *P*<0.05 and P<0.001, respectively; ANOVA). The RCR values were compared using the paired T–test.

We investigated how AgMC1 silencing affected mitochondrial respiration and ROS generation. We have previously shown that increasing the level of ROS in the midgut by silencing genes involved in ROS detoxification such as catalase [9] or oxidation resistance gene 1 (OXR1) [43] reduces *P. berghei* infection. The hypothesis that AgMC1 silencing enhanced infection by reducing mitochondrial ROS generation was investigated. AgMC1 silencing had no effect on ADP-stimulated mitochondrial respiration (state 3) (Fig. 3A). However, state 4 respiration, the phosphorylation-independent oxygen consumption after inhibition F_1_F_0_-ATP synthase with oligomycin, was significantly higher in AgMC1-silenced midguts (*P*<0.05) (Figure 3A). As a consequence, the respiratory control ratio (RCR  =  state 3/state 4 respiration), an index used to assess mitochondrial inner membrane integrity, is significantly lower (*p*<0.005) (Figure 3B), indicating increased proton permeability of the inner mitochondrial membrane. The effect of silencing the *Aedes aegypti* ortholog of AgMC1 (AaMC1) on midgut respiration was also evaluated. As in *A. gambiae*, silencing AaMC1 had no effect on state 3 respiration in *A. aegypti* midguts (Figure 3C), while state 4 respiration was significantly increased (Figure 3C). The RCR was also significantly reduced (*P* = 0.0199) in AaMC1-silenced midguts (Figure 3D), indicating that the role of MC1 in mitochondrial respiration is conserved in anopheline and culicine mosquitoes. Consistent with these findings dsAaMC1-injected mosquitoes had a significantly higher median number of oocysts when infected with *Plasmodium gallinaceum* (*P*<0.007, Mann-Whitney Test)(Figure S2); the difference in the oocyst distributions was marginally significant (*P*<0.07, KS Test) and the prevalence of infection increased significantly from 63% to 80% (*P*<0.008, Chi-square Test).

As MC1 silencing increased proton permeability of the inner mitochondrial membrane, we investigate whether this affected mitochondrial membrane potential. Adult females were fed a solution containing MitoTracker Red, a fluorescent cationic dye that is selectively transported into energized mitochondria. MitoTracker Red uptake was greatly reduced in AgMC1 silenced midguts (Figure 3E), indicating decreased mitochondrial membrane potential, in agreement with the observed reduction in RCR, that is also indicative of mitochondrial uncoupling (Fig. 3B).

**Figure 4:**
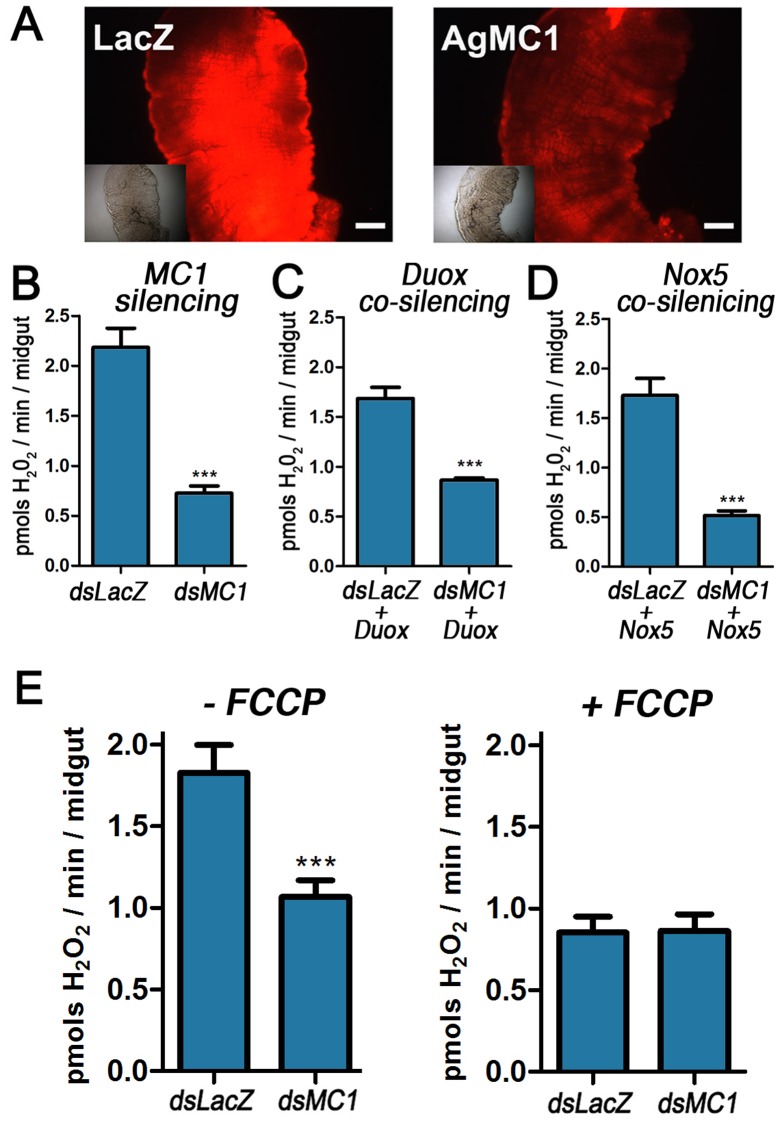
Effect of AgMC1 silencing on mitochondrial ROS generation in the midgut. (A) Control (dsLacZ) and dsAgMC1-silenced midguts from sugar-fed mosquitoes were incubated with dihydroethidine (DHE), a dye that becomes fluorescent in response to ROS. DIC images of the same midguts are shown in the inset. Scale bar  = 100 µm. (B) Effect of AgMC1 silencing, co-silencing Duox (C) or NOX5 (D) or adding FCCP (E) on midgut H_2_O_2_ generation detected using the Amplex red assay. Resorufin fluorescence was measured at 590 nm. Bars represent means ± SE. Significant differences are indicated by the asterisks (***indicates *P*<0.001; Student’s *t*-test).

Because even modest changes in mitochondrial membrane potential can greatly affect ROS generation [22], the effect of AgMC1 silencing on ROS levels was explored. Midguts were incubated with dihydroethidium (DHE), a dye that can be used to monitor ROS *in vivo*, as the products of intracellular DHE oxidation are highly fluorescent [38]. Midguts from AgMC1-silenced mosquitoes consistently exhibit reduced fluorescence when incubated with DHE (Figure 4A), suggesting reduced intracellular ROS. Midgut hydrogen peroxide (H_2_O_2_) production was measured directly using the Amplex Red detection system. Mosquitoes were pre-treated with antibiotics to eliminate the gut microbiota as a potential source of ROS and 3-amino-1,2,4-triazole was included in the reactions to inhibit catalase activity and prevent H_2_O_2_ degradation. AgMC1 was efficiently silenced (Figure S3) and H_2_O_2_ generation was significantly reduced in silenced midguts (*P*<0.0001) (Figure 4B). Furthermore, co-silencing dual oxidase (Duox) (Figure 4C) or NADPH oxidase (Figure 4D), two oxidases that could be potential sources of H_2_O_2_, did not eliminate the differences in H_2_O_2_ levels between control and dsMC1-silenced midguts. Silencing validation for the co-silencing experiments is shown in Figure S4. In contrast, when mitochondria were uncoupled by adding FCCP (Figure 4E), the production of H_2_O_2_ in the dsLacZ group decreased (p<0.001) to levels not significantly different from the AgMC1-silenced group. It is also noteworthy that FCCP had no significant effect on H_2_O_2_ production in midguts in which AgMC1 had been silenced, indicating that midgut mitochondria were already partially uncoupled. Taken together, these data indicate that AgMC1 silencing decreases mitochondrial uncoupling and ROS production in the mosquito midgut.

## Discussion

Mitochondrial ROS generation is regulated by a number of factors such as the oxygen pressure, substrate availability, efficiency of the electron flow and mitochondrial membrane potential; and changes in any of these parameters can potentially modulate the host’s susceptibility to infection. Knockdown of the uncoupling protein 2 (UCP2) in mice increases mitochondrial membrane potential, increases ROS production and confers resistance to Toxoplasma infection [15].

Although the substrate of AgMC1 is unknown, the binding region of the predicted protein structure (Fig. 1E) is lined with charged and polar amino acid chains and lacks an ADP/ATP binding consensus sequence, suggesting that AgMC1 carries a polar substrate other than ADP/ATP. The phylogenetic analysis revealed that AgMC1 and human SLC25A39 and SLC25A40 cluster with the yeast manganese trafficking to mitochondria 1 (MTM1) protein (Fig. 1*A*). Manganese transport by MTM1 has been shown to be important for the enzymatic activity of Mn-SOD [41] in yeast and, in vertebrates, SLC25A39 has been shown to be involved in heme biosynthesis [44]. It is unlikely that AgMC1 is required for *A. gambiae* Mn-SOD to be active, because reducing Mn-SOD activity would increase mitochondrial ROS levels and we found that AgMC1 silencing has the opposite effect, decreasing ROS generation.

The reduced respiratory control ration (RCR) in AgMC1 and AaMC1-silenced midguts (Fig. 3C and D) are due to an increase in state 4 respiration (Fig. 3B), indicating increased proton leakage. The mechanisms that regulate the basal rate of proton leak are not clear, but it is known that mitochondrial carriers, such as ANT [30], [45], [46] and the 2-oxoglutarate transporter [46] can affect mitochondrial coupling. We do not know how AgMC1 affects mitochondrial coupling, but one can speculate that it could act as an ion transporter from the matrix to the intermembrane space, thus increasing the proton motive force. Alternatively, AgMC1 could act as a repressor of uncoupling proteins (UCPs), limiting the proton conductance mediated by these proteins.

Reactive oxygen and nitrogen species (ROS and RNS) are important effectors of the immune system. The mosquito midgut is an important barrier against a variety of pathogens including bacteria and *Plasmodium*, and ROS/RNS production has been shown to limit *Plasmodium* infection in anopheline mosquitoes [6], [8], [9]. Ookinete invasion triggers a nitration response as a two-step process in which NOS induction is followed by a peroxidase-mediated reaction that involves the heme peroxidase 2 (HPX2) and Nox5 as a source of H_2_O_2_ [4], [5], [6]. Recent studies revealed that these reactions can also modify ookinetes as they traverse the midgut, acting as an opsonization-like system that promotes activation of the mosquito complement [6]. This model predicts that ookinete survival would depend on the probability of being “tagged” for destruction as a parasite traverses the midgut. We have previously shown that reducing H_2_O_2_ detoxification by silencing catalase enhances *A. gambiae* resistance to *P. berghei* infection. Conversely, decreasing ROS, by oral administration of antioxidants prevents melanization of *Plasmodium* [8]. Our observation that AgMC1 silencing reduces mitochondrial membrane potential and ROS production in the midgut and decreases resistance to *Plasmodium* infection, indicates that mitochondria ROS can modulate antiplasmodial responses. Lower levels of H_2_O_2_ in the invaded cells are expected to reduce the rate of nitration, increasing the probability that ookinetes escape unharmed and evade detection by the mosquito complement system. Recently, mitochondrial ROS have also been proposed to be important modulators of immunity. For example, ROS generated by mitochondria of endothelial cells in response to hypoxia decreases TLR4 expression [47]. It is possible that reduced mitochondrial ROS generation following AgMC1 silencing is affecting signaling cascades that regulate antiplasmodial responses in mosquitoes.

We explored mitochondrial function in the *A. gambiae* midgut and characterized a novel member of the mitochondrial carrier gene family, AgMC1, that modulates midgut mitochondrial membrane potential and ROS production. Our studies revealed a novel link between mitochondrial metabolism and mosquito epithelial responses to *Plasmodium* invasion.

## Supporting Information

Figure S1 **Sequence alignment of members of the mitochondrial family from different species.** AgMC1 (AGAP001297-PA) and the putative *A. aegypti* (AaMC1, AAEL001329) and *D. melanogaster* (DmMC1, CG14209) putative ortholog genes, human mitochondrial carriers HsSLC25A-39 (NP_057100) and SLC25A-40 (NP_061331), and yeast manganese trafficking factor for mitochondrial (ScMTM1, EDN61842); human uncoupling protein 5 (HsUCP, NP_003942), *A. gambiae* (AgUCP, AGAP011839-PA), *A. aegypti* (AaUCP, AAEL011842) and *D. melanogaster* (DmUCP, CG7314-RB) uncoupling proteins; Human SLC25A6 (HsANT, NP_001142) and yeast (ScANT, AAA97484) adenine nucleotide translocators, *A. gambiae* (AgANT, AGAP006782-PA), *A. aegypti* (AaANT, AAEL004855) and *D. melanogaster* (DmANT, CG16944) putative adenine translocators; human SLC25A3 (HsPiC, NP_005879) and yeast (ScPiC, NP_010973) phosphate carriers, and *A. gambiae* (AgPiC, AGAP003586-PA), *A. aegypti* (AaPiC, AAEL011184) and *D. melanogaster* (DmPiC, CG4994-PA) putative phosphate carriers.
(PDF)

Figure S2 **Effect of AaMC1 silencing on** ***A. aegypti*** **susceptibility to** ***P. gallinaceum*** **infection.** *Aedes aegypti* females were fed *P. gallinaceum* infected blood (5% parasitemia) and the intensity of infection was determined 8 days after feeding using mercurochrome to stain the midguts. Medians are indicated by the red lines and distributions were compared using the Kolmogorov-Smirnov test.
(TIFF)

Figure S3 **Effect of AgMC silencing by systemic injection of dsRNA on AgMC1, AgDuox and AgNox5 midgut mRNA levels.** Significant differences relative to the dsLacZ control are indicated by the asterisks (***indicates *P*<0.001; Student’s t test).
(TIFF)

Figure S4 **Effect of AgMC1 silencing and co-silencing AgDuox or AgNox5 by systemic injection of dsRNA on AgMC1, AgDuox and AgNox5 midgut mRNA levels.** Significant differences are indicated by the asterisks (***indicates P<0.001; Student’s t test).
(TIFF)

Table S1 **Amino acid sequence identity and homology of the predicted AgMC1 protein with other members of the solute carrier family.**
(PDF)
