# Supplementary material for: Mitochondrial Reactive Oxygen Species Modulate Mosquito Susceptibility to Plasmodium Infection
Source: PLoS One. 2012 Jul 18;7(7):e41083. doi: 10.1371/journal.pone.0041083 (PMC3399787; doi:10.1371/journal.pone.0041083)
Supplement: Figure S1 — Sequence alignment of members of the mitochondrial family from different species. AgMC1 (AGAP001297-PA) and the putative A. aegypti (AaMC1, AAEL001329) and D. melanogaster (DmMC1, CG14209) putative ortholog genes, human mitochondrial carriers HsSLC25A-39 (NP_057100) and SLC25A-40 (NP_061331), and yeast manganese trafficking factor for mitochondrial (ScMTM1, EDN61842); human uncoupling protein 5 (HsUCP, NP_003942), A. gambiae (AgUCP, AGAP011839-PA), A. aegypti (AaUCP, AAEL011842) and D. melanogaster (DmUCP, CG7314-RB) uncoupling proteins; Human SLC25A6 (HsANT, NP_001142) and yeast (ScANT, AAA97484) adenine nucleotide translocators, A. gambiae (AgANT, AGAP006782-PA), A. aegypti (AaANT, AAEL004855) and D. melanogaster (DmANT, CG16944) putative adenine translocators; human SLC25A3 (HsPiC, NP_005879) and yeast (ScPiC, NP_010973) phosphate carriers, and A. gambiae (AgPiC, AGAP003586-PA), A. aegypti (AaPiC, AAEL011184) and D. melanogaster (DmPiC, CG4994-PA) putative phosphate carriers. (PDF) [file pone.0041083.s001.pdf]

CLUSTAL W (1.83) multiple sequence alignment

```
AgMC1          ---------------------------------------------------MARSVGS-- 7
AeMC1          ---------------------------------------------------MTAESGSRS 9
DmMC1          -----------------------------------------MATREACGQFAAASAAMAA 19
HsSLC25A40     ------------------------------------------------------------
HsSLC25A39     ------------------------------------------------------------
ScMTM1         ------------------------------------------------------------
AgANT          ------------------------------------------------------------
AeANT          ------------------------------------------------------------
DmANT          ---------------------------------------------------------MGN 3
HsANT          ------------------------------------------------------------
ScANT          ---------------------------------------------------------MSS 3
AgUCP5         ------------------------------------------------------------
AeUCP          ------------------------------------------------------------
DmUCP          ------------------------------------------------------------
HsUCP5         ----------------------------------------MGIFPGIILIFLRVKFATAA 20
AgPiC          MFAGLLDAARNSPFRTPFTRVHCDDGTEKAVVPGRSVQAAASRECDAGTVAPVRGRSIAA 60
AePiC          MFSALLDAARNSPFKTPFTKAQCD--------------AAAPSENPKSLIA--SGRTLAA 44
DmPiC          MFSSFFETARNSPFRTPMSMARCD--------------AAAPVVEPQ----PVEGRQIAA 42
HsPiC          MFSSVAHLARANPFNTPHLQLVHDG------------LGDLRSSSPGPTGQPRRPRNLAA 48
ScPiC1         -----MESNKQP------------------------------------------------ 7


AgMC1          -SVAVAPGEDMDDSRFRIRPYQQILSSCSGALVTSLFMTPLDVVKTRLQAQQKVLISN-- 64
AeMC1          ESPLPAPGVDMDDSRFRIRPYQQILSSCSGALVTSLFMTPLDVVKTRLQTQQKMMLSN-- 67
DmMC1          ASSQNPSKATMTDPRFRIRPLQQVASACTGAMVTACFMTPLDVIKTRLQAQQQALLSN-- 77
HsSLC25A40     -----MDPETRGQEIIKVTPLQQMLASCTGAILTSVIVTPLDVVKIRLQAQNNPLP---- 51
HsSLC25A39     ---------MADQDPAGISPLQQMVASGTGAVVTSLFMTPLDVVKVRLQSQRPSMASELM 51
ScMTM1         --------MSDRNTSNSLTLKERMLSAGAGSVLTSLILTPMDVVRIRLQQQQMIPDCSCD 52
AgANT          ---------MT-KKADPYGFAKDFLAGGISAAVSKTAVAPIERVKLLLQVQ-AASKQIA- 48
AeANT          ---------MSGKKADPYGFAKDFLAGGISAAVSKTAVAPIERVKLLLQVQ-AASKQIA- 49
DmANT          ISASITSQSKMGKDFDAVGFVKDFAAGGISAAVSKTAVAPIERVKLLLQVQ-HISKQIS- 61
HsANT          ------------MGDHAWSFLKDFLAGGVAAAVSKTAVAPIERVKLLLQVQ-HASKQIS- 46
ScANT          NAQVKTPLPPAPAPKKESNFLIDFLMGGVSAAVAKTAASPIERVKLLIQNQDEMIKQGT- 62
AgUCP5         ------------MSDIR--DWRPFVYGGMASIMAEFGTFPIDTTKTRLQIQGQKTDRSH- 45
AeUCP          ------------MGEIR--DWRPFIYGGVASITAEFGTFPIDTTKTRLQIQGQKIDQSH- 45
DmUCP          ------------MGEVK--DWRPFVYGGVASITAEFGTFPIDTTKTRLQIQGQKIDQSF- 45
HsUCP5         VIVSGHQKSTTVSHEMSGLNWKPFVYGGLASIVAEFGTFPVDLTKTRLQVQGQSIDARF- 79
AgPiC          AAT------DEVEFGSTKFFLLCGLGGIISCGSTHTFVVPLDLVKCRLQVD--------- 105
AePiC          AAS------DEVEFGSTQFYLLCGVGGIISCGSTHTFVVPLDLVKCRLQVD--------- 89
DmPiC          AATPVANQQDSCEFGSTKYFALCGIGGILSCGTTHTFVVPLDLVKCRLQVD--------- 93
HsPiC          AAVEEQ---YSCDYGSGRFFILCGLGGIISCGTTHTALVPLDLVKCRMQVD--------- 96
ScPiC1         ---------RKIQLYTKEFYATCTLGGIIACGPTHSSITPLDLVKCRLQVN--------- 49
                                         .  ..  :     *::  :  :* :

AgMC           -------------KCYLYCNG---LMDHICPCGPNGPMAPSTISKRPLHLTGTIDAFTKI 108
AeMC1          -------------KCYLYCNG---LMDHLCPCGPNG----TAFAKPKLHFTGTIDAFVKI 107
DmMC1          -------------KCFLYCNG---LMDHICPCGPDTPN--PAAAKPAPRFSGTIDAFIKI 119
HsSLC25A40     -----------KGKCFVYSNG---LMDHLCVCEEGG---NKLWYKKPGNFQGTLDAFFKI 94
HsSLC25A39     PSSRLWSLSYTKWKCLLYCNG---VLEPLYLCPNGAR--CATWFQDPTRFTGTMDAFVKI 106
ScMTM1         GAAEVPNAVSSGSKMKTFTNVGGQNLNNAKIFWESACFQELHCKNSSLKFNGTLEAFTKI 112
AgANT          ---------------------------------------------VDQQYKGIVDCFVRI 63
AeANT          ---------------------------------------------ADKQYKGIVDCFVRI 64
DmANT          ---------------------------------------------PDKQYKGMVDCFIRI 76
HsANT          ---------------------------------------------AEKQYKGIIDCVVRI 61
ScANT          ---------------------------------------------LDKKYSGIVDCFKRT 77
AgUCP5         ---------------------------------------------SELRYRGMTDAFVKI 60
AeUCP          ---------------------------------------------AELKYRGMTDAFVKI 60
DmUCP          ---------------------------------------------SQLRYRGMTDAFVKI 60
HsUCP5         ---------------------------------------------KEIKYRGMFHALFRI 94
AgPiC          ----------------------------------------------QAKYKNVFHGFKLT 119
AePiC          ----------------------------------------------QAKYKNLFHGFKVS 103
DmPiC          ----------------------------------------------QAKYKNLVHGFKVT 107
HsPiC          ----------------------------------------------PQKYKGIFNGFSVT 110
ScPiC1         ----------------------------------------------PKLYTSNLQGFRKI 63
                                                               .  . .

AgMC1          SRYEGVPSLWSGLGPTLILALPTTVIYFVAYEQFRIRLKELYQRRKGRD--------AEL 160
AeMC1          SHHEGVKSLWSGLSPTLVLALPTTVIYFVAYEQFRLRLKEFYLRKRDKS--------AEL 159
DmMC1          SRTEGIGSLWSGLSPTLISALPSTIIYFVAYEQFKARFTDIHYKYTRRPDTIAHDIPHPI 179
```

```
HsSLC25A40     IRNEGIKSLWSGLPPTLVMAVPATVIYFTCYDQLSALLRSKLGEN--------------- 139
HsSLC25A39     VRHEGTRTLWSGLPATLVMTVPATAIYFTAYDQLKAFLCGRALTS--------------- 151
ScMTM1         ASVEGITSLWRGISLTLLMAIPANMVYFSGYEYIRDVSPIASTYP--------------- 157
AgANT          PKEQGIGAFWRGNLANVIRYFPTQALNF-AFKDVYKQVFLGGVDKNTQF---------WR 113
AeANT          PKEQGFGAFWRGNLANVIRYFPTQALNF-AFKDVYKQIFLGGVDKNTQF---------WR 114
DmANT          PKEQGFSSFWRGNLANVIRYFPTQALNF-AFKDKYKQVFLGGVDKNTQF---------WR 126
HsANT          PKEQGFLSFWRGNLANVIRYFPTQALNF-AFKDKYKQLFLGGVDRHKQF---------WR 111
ScANT          AKQEGLISFWRGNTANVIRYFPTQALNF-AFKDKIKLMF--GFKKEEGY---------GK 125
AgUCP5         SRQEGVKALYSGIWPAVLRQATYGTIKFGTYYTLKKVATDRGLLHDKAG---------NE 111
AeUCP          SKQEGMKALYSGIWPAVLRQATYGTIKFGTYYTLKKVAIEKGWLVDKSG---------NE 111
DmUCP          SREEGLRALYSGIWPAVLRQATYGTIKFGTYYTLKKLANERGLLINEDG---------SE 111
HsUCP5         CKEEGVLALYSGIAPALLRQASYGTIKIGIYQSLKRLFVER--LED-------------E 139
AgPiC          LAEDGSRGLVKGWAPTFFGYSAQGAFKFGLYEVFKVQYANMLGEENAYL----------Y 169
AePiC          VAEEGAKGLVKGWAPTFFGYSAQGAFKFGLYEVFKVQYANVLGEENAYL----------Y 153
DmPiC          VAEEGARGLAKGWFPTLLGYSAQGLCKFGLYELFKVKYAEIIGEENAYL----------Y 157
HsPiC          LKEDGVRGLAKGWAPTFLGYSMQGLCKFGFYEVFKVLYSNMLGEENTYL----------W 160
ScPiC1         IANEGWKKVYTGFGATFVGYSLQGAGKYGGYEYFKHLYSSWLSPG--------------- 108
                  :*   .  *    ..            :

AgMC1          PIWLPLLAGSSARVLAVTIVNPLELIRTKMQSEK---------LSYREVGQAFRSMLRVQ 211
AeMC1          PFWLPLVAGGSARVMAVTIVNPLELIRTKMQSEK---------LSYTEVGRGFRSMLKMQ 210
DmMC1          PFLVPLLAGVSGRILAVTCVSPVELIRTKMQSQR---------MTHAEMFGTIRQVVQSQ 230
HsSLC25A40     ETCIPIVAGIVARFGAVTVISPLELIRTKMQSKK---------FSYVELHRFVSKKVSED 190
HsSLC25A39     DLYAPMVAGALARLGTVTVISPLELMRTKLQAQH---------VSYRELGACVRTAVAQG 202
ScMTM1         -TLNPLFCGAIARVFAATSIAPLELVKTKLQSIPRSSKSTKTWMMVKDLLNETRQEMKMV 216
AgANT          YFLGNLGSGGAAGATSLCFVYPLDFARTRLGADVG---RGAGEREFNGLLDCLKKTVKSD 170
AeANT          YFMGNLGSGGAAGATSLCFVYPLDFARTRLGADVG---RAGAEREYNGLIDCLKKTVKSD 171
DmANT          YFAGNLASGGAAGATSLCFVYPLDFARTRLAADTG---KGG-QREFTGLGNCLTKIFKSD 182
HsANT          YFAGNLASGGAAGATSLCFVYPLDFARTRLAADVG---KGAAQREFHGLGDCIIKIFKSD 168
ScANT          WFAGNLASGGAAGALSLLFVYSLDYARTRLAADSKSS-KKGGARQFNGLIDVYKKTLKSD 184
AgUCP5         SLWCNAACATMAGAISSAIANPTDVLKVRMQVHGR-------GTSDVGLVQCFREIYVHE 164
AeUCP          NVWCNAGCATIAGAVSSAIANPTDVLKVRMQVHGK-------GTNNAGLARCFKEIYVHE 164
DmUCP          RVWSNILCAAAAGAISSAIANPTDVLKVRMQVHGK-------GQ-HKGLLGCFGEIYKYE 163
HsUCP5         TLLINMICGVVSGVISSTIANPTDVLKIRMQAQG--------SLFQGSMIGSFIDIYQQE 191
AgPiC          RTWLYLGASASAEFFADMALSPFEAAKVKIQT-MPG--------FASNMREAMPKMMGEE 220
AePiC          RTWLYLAASASAEFFADIALSPMEAAKVKIQT-MPG--------FAGTLREAMPKMMGEE 204
DmPiC          RTSLYLAASASAEFFADIALAPFEAAKVKIQT-IPG--------YANNFREAVPKMLKEE 208
HsPiC          RTSLYLAASASAEFFADIALAPMEAAKVRIQT-QPG--------YANTLRDAAPKMYKEE 211
ScPiC1         -VTVYLMASATAEFLADIMLCPFEAIKVKQQTTMPP--------FCNNVVDGWKKMYAES 159
                      ..  .   :     . :  : :                   .

AgMC1          G-ILGLWKGFFPTILRDVPFSGIYWTTYESFKKHFN-----------VSQPTFAFSFAGG 259
AeMC1          G-IMGLWKGFFPTILRDVPFSGIYWTTYETFKKRCN-----------VTQPTFGVSFVGG 258
DmMC1          G-VLGLWRGLPPTILRDVPFSGIYWTCYEYLKSSFG-----------VVEPTFSFSFAAG 278
HsSLC25A40     G-WISLWRGWAPTVLRDVPFSAMYWYNYEILKKWLCEKSG-------LYEPTFMINFTSG 242
HsSLC25A39     G-WRSLWLGWGPTALRDVPFSALYWFNYELVKSWLNGFRP-------KDQTSVGMSFVAG 254
ScMTM1         GPSRALFKGLEITLWRDVPFSAIYWSSYELCKERLWLDSTRFA-SKDANWVHFINSFASG 275
AgANT          G-IIGLYRGFNVSVQGIIIYRAAYFGCFDTAKGMLPD----------PKNTSIFVSWAIA 219
AeANT          G-LIGLYRGFNVSVQGIIIYRAAYFGCFDTAKGMLPD----------PKNTSIFVSWAIA 220
DmANT          G-IVGLYRGFGVSVQGIIIYRAAYFGFYDTARGMLPD----------PKNTPIYISWAIA 231
HsANT          G-LRGLYQGFNVSVQGIIIYRAAYFGVYDTAKGMLPD----------PKNVHIFVSWMIA 217
ScANT          G-VAGLYRGFLPSVVGIVVYRGLYFGMYDSLKPLLLTG---------SLEGSFLASFLLG 234
AgUCP5         G-IRGLWRGVGPTAQRAAVIAAVELPVYDFCKLHLMET----------FGDQVANHFISS 213
AeUCP          G-VRGLWRGVGPTAQRAAVIAAVELPVYDFCKLHLMET----------FGDQVANHFISS 213
DmUCP          G-VRGLWRGVGPTAQRAVVIASVELPVYDFCKLQLMNA----------FGDHVGNHFISS 212
HsUCP5         G-TRGLWRGVVPTAQRAAIVVGVELPVYDITKKHLILSG--------MMGDTILTHFVSS 242
AgPiC          G-IMAFYKGLVPLWCRQIPYTMMKFACFEKTVELLYAYVVPKPRDQCSKGEQLIVTFAAG 279
AePiC          G-ITAFYKGLVPLWCRQIPYTMMKFACFEKTVELLYAYVVPKPRDQCSKGEQLLVTFAAG 263
DmPiC          G-VNAFYKGLVPLWMRQIPYTMMKFACFERTVELLYKYVVPKPRADCTKGEQLIVTFAAG 267
HsPiC          G-LKAFYKGVAPLWMRQIPYTMMKFACFERTVEALYKFVVPKPRSECSKPEQLVVTFVAG 270
ScPiC1         GGMKAFYKGIVPLWCRQIPYTMCKFTSFEKIVQKIYS-VLPKKKEEMNALQQISVSFVGG 218
               *   .:: *                  ::                       .   :  .

AgMC1          AISGGVAAFFTVPFDVVKTHQQIAFGEQFLYAQNGDSKA-------------------AG 300
AeMC1          AISGGVAAFLTVPFDVVKTHQQIEFGEKFLYAENGEK----------------------- 295
DmMC1          AISGSVAATITTPFDVVKTHEQIEFGEKFIFSDNPPK----------------------- 315
HsSLC25A40     ALSGSFAAVATLPFDVVKTQKQTQLWT---YESHKISM---------------------- 277
HsSLC25A39     GISGTVAAVLTLPFDVVKTQRQVALGA---MEAVRVN----------------------- 288
ScMTM1         CISGMIAAICTHPFDVGKTRWQISMMN----NSDPKG----------------------- 308
AgANT          QVVTTASGIISYPFDTVRRRMMMQSGRA-------------------------------- 247
AeANT          QVVTTASGVISYPFDTVRRRMMMQSGRA-------------------------------- 248
DmANT          QVVTTVAGIVSYPFDTVRRRMMMQSGRK-------------------------------- 259
HsANT          QSVTAVAGLVSYPFDTVRRRMMMQSGRK-------------------------------- 245
```

```
ScANT          WVVTTGASTCSYPLDTVRRRMMMTSG---------------------------------- 260
AgUCP5         FIASLGSAIASTPIDVIRTRLMNQRRVHQLQPSITP-----------------AATTTTT 256
AeUCP          FIASLGSAVASTPIDVIRTRLMNQRRVQLQVHNLGPGGGGGGGGRGVAGGGGLAAVANPS 273
DmUCP          FIASLGSAIASTPIDVIRTRLMNQRPVSITMN---------------------GVVTAAA 251
HsUCP5         FTCGLAGALASNPVDVVRTRMMNQRAIVGHVD---------------------------- 274
AgPiC          YIAGVFCAIVSHPADVVVSKLNQAKGS--------------------------------- 306
AePiC          YIAGVFCAIVSHPADVVVSKLNQAKGS--------------------------------- 290
DmPiC          YIAGVFCAVVSHPADVVVSKLNQAKGA--------------------------------- 294
HsPiC          YIAGVFCAIVSHPADSVVSVLNKEKGS--------------------------------- 297
ScPiC1         YLAGILCAAVSHPADVMVSKINSERKAN-------------------------------- 246
                      .  : * *

AgMC1          PKKPVRSIGTFETMGRIFQMNGIRGLFAGLTPRLVKVAPACAIMIASFEYGKNFFYRYNV 360
AeMC1          -KKPQKSSGTFETMRNIYVRNGIKGLFAGLTPRLVKVAPACAIMIASFEYGKNFFYNYNV 354
DmMC1          ---QVATKSVAMRLASIYRMGGVPAIFSGLGPRLFKVAPACAIMISSFEYGKSFFYHYNI 372
HsSLC25A40     ---PLHMS-TWIIMKNIVAKNGFSGLFSGLIPRLIKIAPACAIMISTYEFGKAFFQKQNV 333
HsSLC25A39     ---PLHVDSTWLLLRRIRAESGTKGLFAGFLPRIIKAAPSCAIMISTYEFGKSFFQRLNQ 345
ScMTM1         ---GNRSRNMFKFLETIWRTEGLAALYTGLAARVIKIAPSCAIMISSYEISKKVFGNKLH 365
AgANT          -KSEVMYKNTLDCWVKIAKQEGSGAFFKGAFSNVLR-GTGGALVLVFYDEVKALLG---- 301
AeANT          -KSEIMYKNTLDCWVKIGKTEGSSAFFKGAFSNVLR-GTGGALVLVFYDEVKALMG---- 302
DmANT          -ATEVIYKNTLHCWATIAKQEGTGAFFKGAFSNILR-GTGGAFVLVLYDEIKKVL----- 312
HsANT          -GADIMYTGTVDCWRKIAKDEGAKAFFKGAWSNVLR-GMGGAFVLVLYDEIKKYV----- 298
ScANT          --QAVKYDGAFDCLRKIVAAEGVGSLFKGCGANILR-GVAGAGVISMYDQLQMILFGKKF 317
AgUCP5         TTPRLYYTGSVDCAVQTVRNEGFRALYKGFIPTWVRMGPWNIIFFITYEQLKQFY----- 311
AeUCP          SPSAKIYTGSLDCAIQTVRNEGFRALYKGFIPTWVRMGPWNIIFFITYEQLKQMY----- 328
DmUCP          TP--KLYSGSLDCAVQTIRNEGLPALYKGFIPTWVRMGPWNIIFFITYEQLKKY------ 303
HsUCP5         -----LYKGTVDGILKMWKHEGFFALYKGFWPNWLRLGPWNIIFFITYEQLKRLQI---- 325
AgPiC          ------------SAIDVAKQLGFMGMWNGLMPRIIMIGTLTALQWFIYDGVKVALSIPRP 354
AePiC          ------------SAFDVAKQLGFMGMWNGLMPRIIMIGTLTALQWFIYDGVKVALNIPRP 338
DmPiC          ------------SAISVAKSLGFSGMWNGLTPRIIMIGTLTALQWFIYDGVKVALGIPRP 342
HsPiC          ------------SASLVLKRLGFKGVWKGLFARIIMIGTLTALQWFIYDSVKVYFRLPRP 345
ScPiC1         -------ESMSVASKRIYQKIGFTGLWNGLMVRIVMIGTLTSFQWLIYDSF--------- 290
                                 *  ..: *     .  .         ::


AgMC1          QRYQERKDRVASGSGDGSVVSSSSSTS--- 387
AeMC1          TRYQARWE-----KDQGALIKTKKHTGVGY 379
DmMC1          DQHNRSNQ---ATKGPGS------------ 387
HsSLC25A40     RRQQY------------------------- 338
HsSLC25A39     DRLLGG------------------------ 351
ScMTM1         Q----------------------------- 366
AgANT          ------------------------------
AeANT          ------------------------------
DmANT          ------------------------------
HsANT          ------------------------------
ScANT          K----------------------------- 318
AgUCP5         ------------------------------
AeUCP          ------------------------------
DmUCP          ------------------------------
HsUCP5         ------------------------------
AgPiC          PPPEMPESLKKKLGVQ-------------- 370
AePiC          PPPEMPESLKKKLGVQ-------------- 354
DmPiC          PPPEMPASLKAKQH---------------- 356
HsPiC          PPPEMPESLKKKLGLTQ------------- 362
ScPiC1         ------------------------------
```
