# Supplementary figures and images for: Mitochondrial Reactive Oxygen Species Modulate Mosquito Susceptibility to Plasmodium Infection
Source: PLoS One. 2012 Jul 18;7(7):e41083. doi: 10.1371/journal.pone.0041083 (PMC3399787; doi:10.1371/journal.pone.0041083)

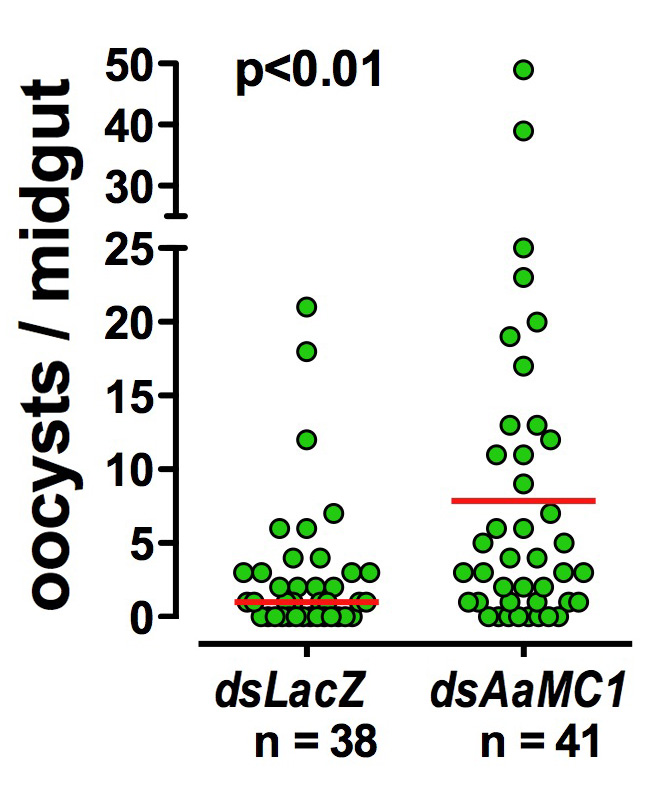

Supplement: Figure S2 — Effect of AaMC1 silencing on A. aegypti susceptibility to P. gallinaceum infection. Aedes aegypti females were fed P. gallinaceum infected blood (5% parasitemia) and the intensity of infection was determined 8 days after feeding using mercurochrome to stain the midguts. Medians are indicated by the red lines and distributions were compared using the Kolmogorov-Smirnov test. (TIFF) [file pone.0041083.s002.tiff]

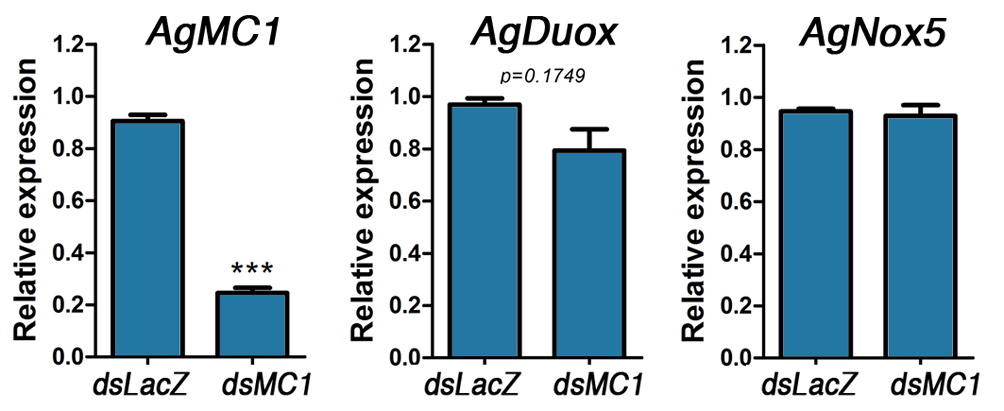

Supplement: Figure S3 — Effect of AgMC silencing by systemic injection of dsRNA on AgMC1, AgDuox and AgNox5 midgut mRNA levels. Significant differences relative to the dsLacZ control are indicated by the asterisks (***indicates P<0.001; Student’s t test). (TIFF) [file pone.0041083.s003.tiff]

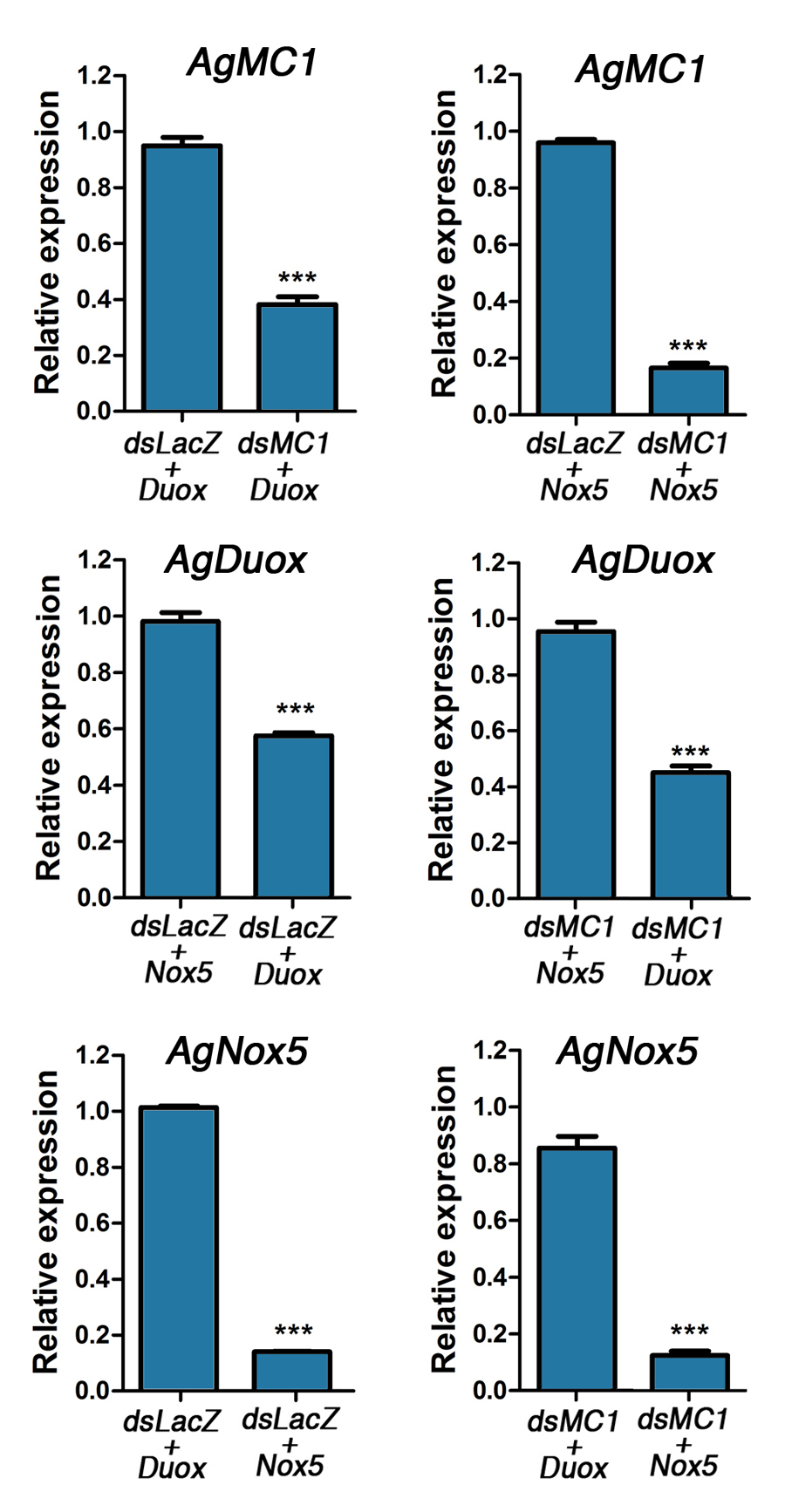

Supplement: Figure S4 — Effect of AgMC1 silencing and co-silencing AgDuox or AgNox5 by systemic injection of dsRNA on AgMC1, AgDuox and AgNox5 midgut mRNA levels. Significant differences are indicated by the asterisks (***indicates P<0.001; Student’s t test). (TIFF) [file pone.0041083.s004.tiff]
