# Supplementary material for: Mitochondrial Reactive Oxygen Species Modulate Mosquito Susceptibility to Plasmodium Infection
Source: PLoS One. 2012 Jul 18;7(7):e41083. doi: 10.1371/journal.pone.0041083 (PMC3399787; doi:10.1371/journal.pone.0041083)
Supplement: Table S1 — Amino acid sequence identity and homology of the predicted AgMC1 protein with other members of the solute carrier family. (PDF) [file pone.0041083.s005.pdf]

Gene Name	Accession No.	Identity	Homology
AeMC1	AAEL001329	79%	87%
AeMC1	AAEL001329	79%	87%
DmMC1	NP_001027071	57%	72%
DmMC1	NP_001027071	57%	72%
HsSLC25A40	NP_061331.2	47%	64%
HsSLC25A39	NP_057100	44%	60%
ScMTM1	EDN61842	34%	48%
DmUCP	CG7314-RB	25%	41%
DmANT	CG16944	24%	44%
AeUCP	AAEL011842	24%	39%
AgUCP5	AGAP011839-PA	24%	38%
ScPiC1	NP_010973	24%	38%
HsPiC	NP_005879	23%	45%
ScANT	AAA97484	23%	45%
AePiC	AAEL011184	23%	43%
HsUCP5	NP_003942	23%	41%
AgPiC	AGAP003586-PA	23%	37%
HsANT	NP_001142	22%	42%
AgANT	AGAP006782-PA	21%	43%
DmPiC	CG4994-PA	21%	35%
AeANT	AAEL004855	20%	43%
